# Strengthening the policy, implementation, and accountability environment for quality care: experiences from quality of care network countries

**DOI:** 10.3389/frhs.2023.1292510

**Published:** 2024-01-16

**Authors:** Blerta Maliqi, Olive Cocoman, Martin Dohlsten, Selina Dussey, Rachael Hinton, Margaret T. Mannah, Moise Muzigaba, Tala Rammal, Jesca Sabiiti, Nuhu Yaqub, Anshu Banerjee

**Affiliations:** ^1^ Department of Maternal, Newborn, Child, Adolescent Health and Aging, World Health Organization, Geneva, Switzerland; ^2^ Quality Management Unit, Ministry of Health, Accra, Ghana; ^3^ Independent Consultant, Geneva, Switzerland; ^4^ National Quality Management Programme, Ministry of Health and Sanitation, Freetown, Sierra Leone; ^5^ Department of Reproductive and Child Health, Ministry of Health, Kampala, Uganda

**Keywords:** policy, quality of care, maternal and child health, health systems, implementation

## Abstract

Despite global commitment to universal health coverage with quality, poor quality of care (QOC) continues to impact health outcomes for mothers and newborns, especially in low-and-middle income countries. Although there is much experience from small-scale projects, without a long-term perspective it is unclear how to implement quality of care effectively and consistently for impact. In 2017, ten countries together with the WHO and a coalition of partners established the Network for Improving Quality of Care for Maternal, Newborn and Child Health (the Network). The Network agreed to pursue four strategic objectives—Leadership, Action, Learning and Accountability (LALA) for QOC. This paper describes, analyses and reflects on what has worked and some of the challenges faced in implementation of the LALA framework. The implementation of the LALA framework has served as a catalyst to develop an enabling environment for QOC in the Network countries through strengthening the policy, implementation, accountability and community engagement for quality care. Developing an enabling health system environment takes time, but it is possible and shows results. The implementation shows that health systems continue to face persistent challenges such as capacities to quickly scale up changes across subnational levels, limited workforce capability to implement quality improvement consistently and gaps in quality of relevant data. The implementation has also highlighted the need to develop new mechanisms for community engagement and learning systems that inform scaling up of good QOC practices across programmes and levels of care. Moving forward, the Network countries will build on the experiences and lessons learned and continue to strengthen the implementation of LALA strategic objectives for impact. We hope the Network experience will encourage other countries and partners to adopt the Network implementation model to enable delivery of quality care for everyone, everywhere, and actively collaborate and contribute to the QOC global learning network.

## Introduction

Globally, lack of quality of care (QOC) continues to impact health outcomes for mothers and newborns (1), especially in low-and-middle income countries (LMICs), where 60% of neonatal conditions and half of maternal deaths are due to poor quality services (1). Resolutions and reports by the World Health Organization (WHO) and the United Nations have called for urgent action on universal health coverage with quality and to strengthen primary healthcare (PHC) (2–4).

Efforts to improve QOC—care that is effective, safe, people centred, timely, equitable, integrated, and efficient (5)—are often project-based and not integrated within broader efforts to strengthen health systems (6). Such projects result in fragmented and disjointed healthcare delivery and have a poor record of sustainability (6). While individual countries and the global community have much experience of what does and does not work to improve quality from small-scale projects, without a long-term perspective we are left without an answer to how to implement QOC effectively and consistently for impact (7).

In 2017, Bangladesh, Côte d'Ivoire, Ethiopia, Ghana, India, Malawi, Nigeria, Sierra Leone, Uganda, and the United Republic of Tanzania, and later Sierra Leone—together with the WHO and a coalition of partners, agreed to establish the Network for Improving Quality of Care for Maternal, Newborn and Child Health (the Network). These countries were identified and engaged based on their demonstrated commitment to QOC, motivation to build the policy and implementation environment for quality, including willingness to openly share data and knowledge (8, 9). The Network provided an opportunity for the countries and partners to form global, regional and national alliances and generate and share learnings to address challenges to QOC implementation for maternal and newborn health (MNH) (8, 9).

At the 2017 launch of the Network at a global meeting in Malawi, over 300 participants from each country's Ministry of Health (MOH), along with members of national and global professional associations and development partners co-developed, deliberated and agreed on the Network vision and strategic objectives. The vision was that every pregnant woman and newborn infant should receive good-quality, and equitable and dignified care throughout pregnancy, childbirth and the postnatal period (8, 10). The Network aimed to half institutional maternal and newborn deaths in participating facilities in five years. In working towards this vision, Network countries and partners agreed to pursue four strategic objectives—Leadership: Build and strengthen national institutions and mechanisms for improving QOC in the health sector; Action: Accelerate and sustain implementation of QOC improvements for mothers and newborns; Learning: Facilitate learning, share knowledge and generate evidence on QOC; Accountability: Develop, strengthen and sustain institutions and mechanisms for accountability for QOC (8, 9).

**Figure 1:**
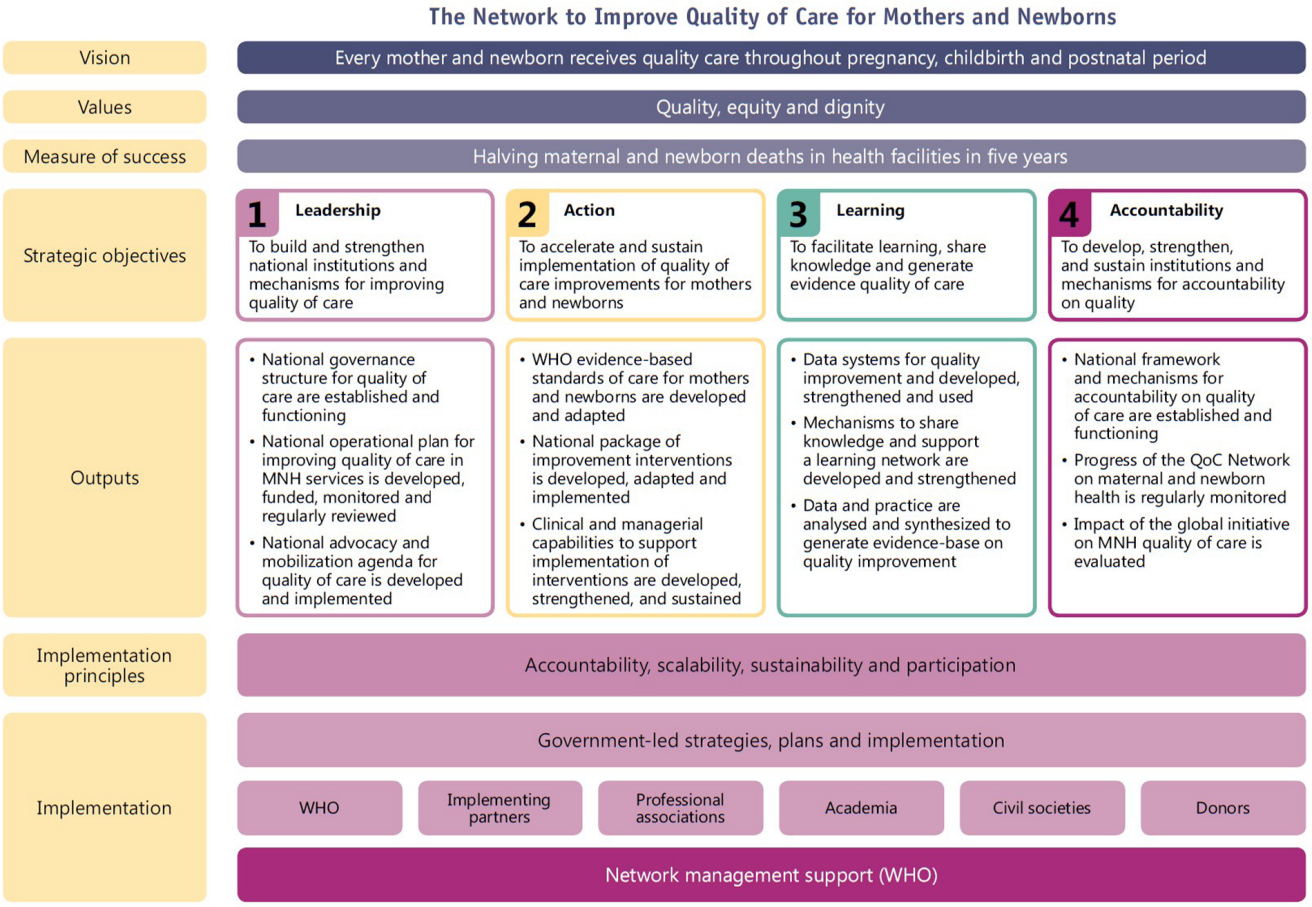
Logical framework of the quality of care network (8).

As the logical framework in Figure 1 shows, the Leadership, Action, Learning and Accountability (LALA) strategic objectives specified outputs needed to operationalise QOC programmes (8). These outputs included planning for and implementing MNH QOC at the national, sub-national and facility levels, monitoring of QOC implementation and impact, and establishing and maintaining a learning system (8). The framework emphasized the need for a national strategic direction on quality health services, and uses MNH as an entry point to demonstrate impact.

The stewarding of the Network is undertaken by the Network Leadership group, a light-touch governance structure, composed of the QOC and MNH leaders in the MOHs from the Network countries. A WHO-based Network secretariat was established to convene, support and coordinate activities across countries and partners, and across WHO (8). The secretariat led the development of the Network implementation guidance and QOC measurement agenda, as well as established and managed a QOC learning network across countries and partners. Global, regional and national partners also played a very important role in the development and implementation of the Network. Partners provided technical and financial support to country implementation, co-developed Network technical guidance and tools, and supported the sharing of learning on QOC implementation. WHO Country Offices worked alongside the MOHs to facilitate the convening of national stakeholders, provide technical assistance, and monitor implementation (see Figure 1) (8).

## Methods for our analysis

This paper is based on the information collated, analysed and synthesised by the Secretariat and Network countries. The Network countries co-developed the LALA strategic objectives framework and reported annually to the Secretariat on progress made in its implementation. This reporting process was supported by semi-annual meetings between the Secretariat and each Network country technical working group composed of MOH QOC leadership and QOC stakeholders and partners, as well as the annual Network global meeting. Other periodic regional and national meetings and workshops, including the national QOC forums contributed to this reporting process. In this paper we describe, analyse and reflect on what has worked and some of the challenges faced in implementation of the LALA framework. We also suggest what needs to be done to further strengthen QOC implementation. We hope by sharing these practices, we can inform other countries and partners that aspire to adopt the Network implementation model, to commit to deliver QOC for everyone, everywhere.

## Reflections from implementation

### Leadership: strengthening governance and creating leadership for improving quality of care

Leadership is defined in the LALA framework as the processes that countries take to build and strengthen national governance structures and mechanisms for improving QOC (8).

Strengthening or establishing leadership for QOC involved the engagement of all parts of the MOH and QOC partners beyond just the MNH programmes. The engagement process motivated countries to establish or strengthen existing QOC departments and pro-actively engage or create multistakeholder working groups. These working groups brought together partners and MOH departments whose work impacted quality of health services for MNH and beyond. Strengthening leadership and governance for quality depended on the creating similar structures at sub-national level. In Ethiopia and Sierra Leone, QOC officer positions were established in the regional bureaus and district; or as in the case of Uganda, terms of references for existing positions were updated to include quality improvement (QI) functions as a responsibility. In some countries, the initial lack of clarity of roles and division of responsibilities between MOH QOC departments and MNH programmes, contributed to tensions on who led the QOC implementation.

Strategic roadmaps for improving MNH QOC were developed to inform implementation of QOC for MNH. The roadmaps considered the existing strengths and weaknesses in the health system in identifying the priorities to accelerate QOC. These documents contained clear milestones, targets, mapping of partners and identification of responsible parties for implementation and monitoring. Within the roadmaps, the development of national quality policy and strategy (NQPS) was identified as a critical milestone necessary for creating the health systems' enabling QOC environment. In 2017, Ethiopia and Ghana had already developed their NQPS and over the course of next five years other countries followed suit (7).

To operationalise the implementation of the MNH QOC roadmaps, countries like Nigeria, developed sub-national operational plans. However, not all countries consistently developed or implemented sub-national plans. This often meant that sub-national implementers were unable to apply the QOC guidance into their day-to-day activities and monitoring, to mobilise and align partners and resources needed to support the QOC implementation, and to participate in QOC learning.

### Action: accelerating and sustaining implementation for quality improvement for MNH

The LALA strategic objective on Action, focuses on the development or adaptation and implementation of QOC standards and interventions, and QOC supporting activities to enhance provision of QOC such as QI. All Network countries developed or updated their national QOC standards for MNH to reflect the WHO QOC MNH standards (11). Countries identified QOC interventions to enable the health system and health workers to deliver QOC for MNH (12). As per country reports to the Network secretariat, Ghana and Nigeria adapted the national quality MNH standards to their sub-national level (regions and states). Following the adaptation, teams at the facility and state/regional levels were trained in QI and in the application of the MNH quality of care standards. District health management teams supported implementation of the standards through regular QI coaching. Findings from QI projects were used to inform development of targeted clinical mentoring. Similarly, QI data and findings were fed into periodic performance reviews and other decision-making processes.

Countries like Bangladesh, Ethiopia, India, and Uganda, that already had capacities and partners involved in supporting QOC for MNH at the sub-national level were quick to start activities and show results. Others faced more difficulties and only started implementation in the third year of the Network. As most Network countries operate in resource constrained systems, these delays were often due to lack of MOH QOC capacities and resources to organize and sustain support to QOC implementation. For example, most countries relied on partners' support to orient and train health care workers in QI or other QOC related interventions (13–16). Implementation at the district and facility levels slowed down during the COVID-19 pandemic but picked up once countries made essential health services an integral part of their response (17–20). The pandemic contributed to disruptions in implementation of MNH QOC plans and activities and the shifting of priorities which lead to the redeployment of resources to support the COVID-19 specific responses. However, some countries such as Ghana, Ethiopia and Nigeria, reported that nascently developed QI capacity through MNH QOC programmes was particularly helpful at the subnational level and in health facilities to support the implementation of infection, prevention and control interventions as well as in their quality control response to reduce COVID-19 transmission.

### Learning: facilitate learning, share knowledge, and generate evidence on QOC

Learning represents the set of actions required to facilitate identification, documentation and sharing of QOC best practices. It also facilitates use of data and knowledge generated to inform decision-making processes aiming to sustain and scale up QOC implementation.

Each Network country activated learning platforms appropriate for their national, sub-national and facility context. These platforms use in-person or virtual peer-to-peer learning, QI collaboratives, communities of practice, etc. to facilitate exchanges within and between QOC teams in facilities and districts (12). For example, Uganda has implemented a QI Collaborative in 171 health facilities in 66 districts to share lessons learned and best practices in implementing QOC MNH, with a focus on pre-term baby care, post-partum haemorrhage of the newborn and the availability of drugs and supplies. Ghana has implemented the QOC activities in 186 primary health facilities in all 16 regions of Ghana. At the national level, Sierra Leone, Ethiopia, Malawi, Uganda, and Ghana have established annual cycles of national QOC learning forums. The forums have a wide-range, multi-sectoral attendance, spanning from health facilities QI teams to high-level government officials. They are used to share best practices, challenges, and innovations to the implementation of QOC policy, strategy and interventions, and to inform scaling up QOC. For example, the Malawi 2022 National QOC Forum was used to reflect on the overall implementation and assess whether the goals of the National Quality Management Policy (2018–2022) were met and agree on next steps and plans for scale up (21). In addition to an annual learning forum, Ghana developed subnational learning forums and publishes semi-annual learning bulletins to further stimulate and reward learning in facilities and districts (22–24).

The impact of QOC learning platforms in scaling up best practices depends on the linkages established between QOC data and practice-based knowledge generation, with programme management processes and policymaking. Learning within and across facilities and districts in the Network countries, happened only after the QOC activities were documented, and facilities used data to demonstrate lessons learned. Similarly, at the national level the best practices and lessons learned were shared only when documented and supported by data. As this new way of working is based on data and documentation generated by practitioners, additional support is needed to improve data quality, documentation of best practices and facilitate the policy dialogue with decision makers. National academic or research institutions are well placed to strengthen these processes in support of QOC scale up.

### Accountability: develop, strengthen, and sustain institutions and mechanisms for accountability for QOC

Demanding and demonstrating accountability are at the centre of the fourth LALA strategic objective. The QOC Network identified community engagement as critical to QOC accountability, especially for voicing and prioritizing QOC concerns and monitoring performance. Community engagement for QOC in the Network countries is still evolving. A national-led vision and strategy to institutionalise community engagement as a core health system activity has been taken forward by Ghana and Malawi. Ghana's community engagement strategy standardizes the structures and processes of the engagement across all regions to develop and implement QOC community scorecards for maternal, newborn and child health, respond to the concerns raised by the community at the facility level across the region and refine QOC implementation (25–27). Malawi established community engagement in the national QOC standards, and mechanisms for the systematic integration of community engagement in the QOC planning and implementation cycle, using tools such as Hospital Ombudsman and health facility committees (21).

Other countries have developed engagement mechanisms that need to be implemented and scaled up. Uganda developed community dialogue guidelines to facilitate community engagement on the findings of quality care assessments (28). However, there is limited documented practice on how to institutionalise community engagement for QOC. Across countries, many community engagement activities remain partner-led and funded projects and struggle to scale up beyond the project scope. Documented learning from Network countries would be critical to identify lessons on community engagement for QOC that can be shared with other countries for adaptation or use.

### Measurement, data and information for leadership, action, learning and accountability

The Network monitoring framework was developed to help countries in the Network track and assess progress towards country-specific and Network-wide goals as well as monitor their improvement efforts across levels of the health system. At the launch of the Network in 2017, countries and stakeholders identified and agreed on essential QOC measurement components which would inform or drive activities around LALA across all countries. The resulting monitoring framework encompasses four QOC measurement and monitoring components: (i) common MNH QOC indicators; (ii) Quality improvement indicators; (iii) Sub-national performance indicators and (iv) Implementation milestones. Most Network countries adapted this framework to develop the MNH QOC agenda as part of their health information systems (HIS).

QOC measurement and monitoring activities at the country level focused on maximizing existing HIS structures, mechanisms, and resources. In most countries, the introduction of new common MNH QOC indicators in national health HIS took longer than was initially expected. The process required in-depth analysis of HIS to map the availability of indicator data elements across different sources such as national indicator reference manuals, DHIS2, summary forms, health facility registers, and individual patient records, etc. Some of the required data elements existed in these sources but were not reported. If new indicators and their metadata had to be added, the process took time to complete due to several factors including timing of HIS review as defined by the national policy, financial resources required for adaptations; capacity building across levels; reporting burden; and negotiations between programme and HIS stakeholders in MOH and partner institutions. WASH and experience of care indicators were generally difficult to collect and report on across the Network. Usually, they are not part of HIS and require specific exit interviews or periodic surveys. By 2023, Sierra Leone, Malawi, Nigeria, and Ghana had integrated most common MNH QOC indicators in their national HIS.

To date, the programme data reported by countries remains limited and is not sufficient and consistent to demonstrate impact across all countries. Limiting factors include challenges and delays in setting up QOC monitoring systems, lack of synchronisation between rolling out QOC activities and monitoring and low and fluctuating number of facilities and districts engaged in active data collection and sharing. By the end of 2022, 195 facilities representing 55 districts in nine countries had shared their QOC common indicators data as part of global Network accountability and learning agenda. On average, Institutional Stillbirth Rate in Ethiopia, Ghana, Nigeria, and Tanzania decreased from the pre- to post-intervention but increased in Malawi, Sierra Leone, and Uganda although not statistically significant. Pre-discharge Neonatal Mortality Rate on the other hand decreased for Ethiopia, Malawi, Sierra Leone, and United Republic of Tanzania but the rates increased for Ghana and Uganda and Obstetric Case Fatality Rate decreased for Sierra Leone and increased for Ethiopia and Malawi (29). However, many health facilities that set up a variety of point of care QI activities were able to see positive results. These activities varied between health facilities and across countries and were aimed at addressing specific quality gaps within the health facility whether directly linked to the common indicators or not.

**Figure 2:**
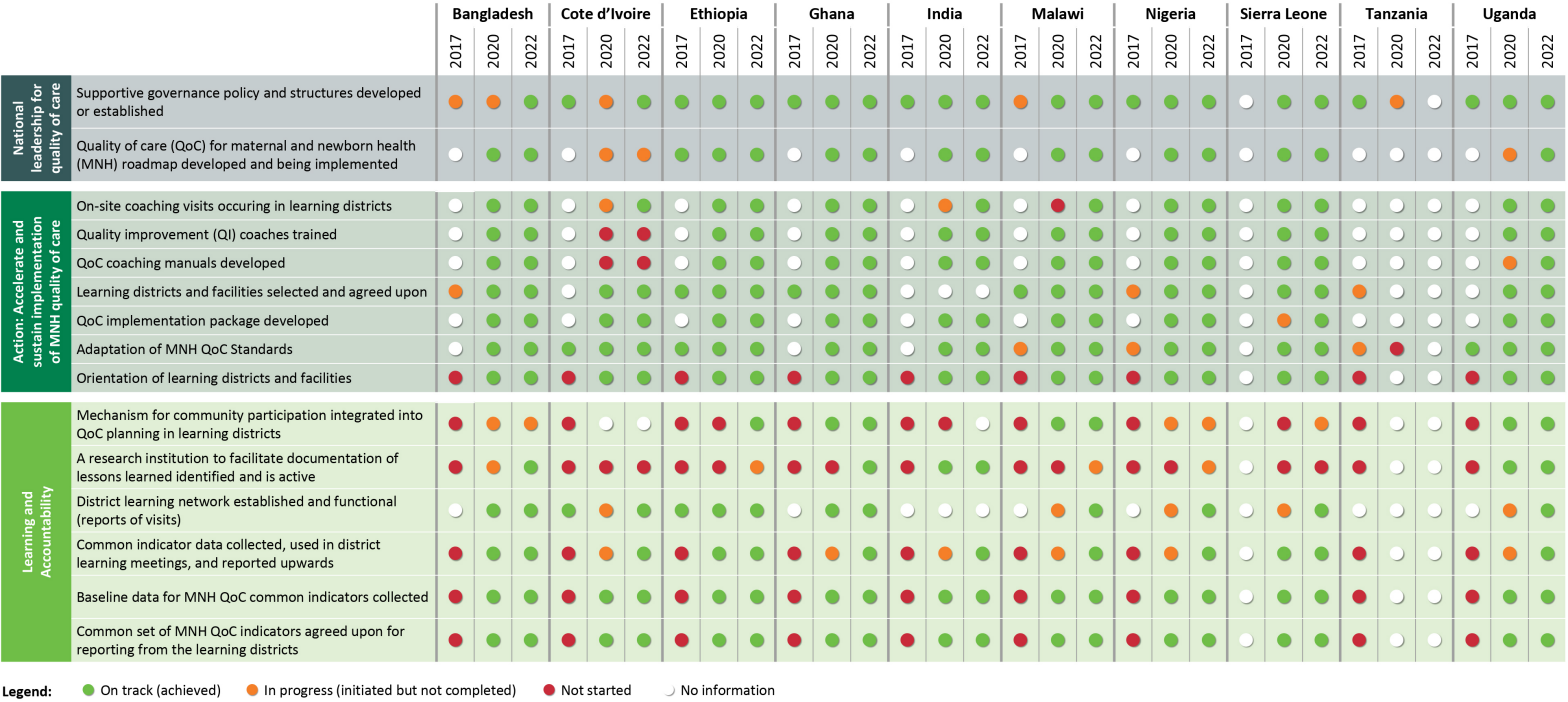
Dashboard of country progress by network strategic objectives, 2017 to 2022.

The Figure 2 presents the dashboard that summarises the progress made by countries in implementing the LALA strategic objectives and milestones (2017–2022). In the dashboard, green indicates that activities towards achievement of the output have been completed; Orange indicates that activities towards achievement of the output have been initiated but have not been completed; Red indicates that activities towards achievement of the output have not been initiated as per plan; White indicates there is no report on progress towards the output.

## Discussion

The Network served as a catalyst for mutual learning on how to strengthen the enabling environment for quality care within and across countries. It stimulated governments and other stakeholders to come together and jointly develop a common vision for quality. Using MNH as an entry point to link quality with impact, countries have strengthened their broader policy and accountability environment for quality care (30). Cocoman and colleagues show that by building QOC structures from national, to sub-national and facility levels, starting with MOH, countries are in a stronger position to continue to guide and strengthen the QOC (31). However, these structures are still very new and need to be strengthened especially at the sub-national level. Countries that simultaneously built capacity and structures at national and sub-national level tended to progress quicker than others (32).

The development and implementation of the Network activities are informed by the principles of the Paris Declaration which call for alignment and harmonisation of partners support around one national plan to improve health systems' effectiveness and accelerate results (33). However, as found by the independent review of the Network in 2022, despite strong national leadership and partners' alignment, it takes time to show results across all levels of care (32, 34).

Unsurprisingly, the efforts to implement and scale up QOC in Network countries was hindered by recurrent gaps in resources and infrastructure (1). High-level turnover of leadership and staff at the different levels of the health system disrupted the commitment and expertise for QOC implementation (32). Capacity and resource gaps were further exacerbated during Covid-19 leading to disruptions in the availability and quality of essential health services, and ultimately poorer health outcomes (20). Countries are now looking to expand the experience developed in improving QOC beyond MNH to other areas such as child health, HIV, nutrition, etc (32). As part of these efforts, they are considering ways to further strengthen the QOC capacities of the workforce beyond MNH by introducing for example capacity building on QI in in-service and pre-service trainings (35). Such capacity will also be central to maintaining coverage of good quality essential health services during public health emergencies (20).

Agweyu et al. highlight that linkages between QOC monitoring and iterative QI at facility, sub-national and national levels must be established (36). In reflection, the Network countries maximized the use of the health information system to monitor QOC, and promote regular use of data in QI processes. These linkages have ensured that made measurement is integrated as a meaningful component of QOC management and learning. Implementation of quality related interventions such as maternal and perinatal death review and response, demonstrate that QOC indicators must be tailored to the needs of key actors across system levels and these actors must have the capacity and enabling environment to collect, analyse and use data on QOC to inform actions to improve care (37).

At the national, and especially sub-national level, there is a need to systemize the sharing and documentation of learning on quality, that so far remains inconsistent and informal. Countries will require technical support to standardise documentation for systematic learning to create a local evidence base on what works and needs to be scaled up in their context (32, 34). National academic or research institutions can be mobilised to support this learning and to align their implementation research efforts to the needs for documenting and informing QOC scale up (38).

Health care users need to be continuously engaged in the QOC dialogue. The Network countries have demonstrated that this can happen when there is a deliberate process and platforms for engaging them (32, 39). However, we are still at the early stages of understanding the mechanisms needed to sustain this engagement. Countries such as Malawi and Ghana have set up structures to strengthen the engagement of communities, but the functionality of these structures has been a challenge, and require further attention to be fully operational and sustained (39).

Moving forward, the Network countries and partners have committed to continue their efforts to scale up the implementation of QOC across programmes and levels of care (35). They will continue to develop and implement national and sub-national QOC policies, strategies and plans integrated within and in support of health strategies and plans. These efforts would maximise use of resources to strengthen health systems capacities; develop QI capabilities for all relevant health worker cadres; strengthen QOC monitoring and data; and develop vibrant national learning systems that bring together implementers, researchers and decision-makers.

Throughout implementation, the WHO-based Network secretariat developed and coordinated a learning network that stimulated and energized a practice-based national, regional and global agenda on QOC for MNH. The secretariat engaged with countries and partners to synthesise countries' needs and experiences. These findings were used for the co-development of global guidance and technical resources, including: the MNH QOC monitoring framework, the QOC implementation guide; the guide for integrating stakeholder and community engagement in QOC; guidance on national learning systems; and knowledge briefs on the implementation of maternal and perinatal death surveillance and the five functions to improve QOC for maternal, newborn and child health (40–45). Seed funding and partners alignment to the Network agenda enabled the operations of the Network at the global and country level. Because the secretariat played a simultaneous role of knowledge broker and information sharing hub, the Network was able to catalyse QOC mutual learning well beyond the Network countries and partners. This has led to demands by other countries to adopt and implement the Network LALA framework in their own context, as well as demands to expand the Network focus to include other programmatic tracers such as QOC for child health.

## Conclusion

The LALA framework has served as a catalyst to develop an enabling environment for QOC in the Network countries through strengthening the policy, implementation, accountability and community engagement for quality care. The Network experience has shown that developing an enabling health system environment to sustain and scale up implementation of quality may take time, but it is possible and shows results. It has also shown that government leadership and commitment, partners' alignment and a systems' approach to complement delivery of QOC is needed to shift from small scale QI projects, to large scale improvements. Data systems and measurement for QOC, along with workforce capacity to improve quality continue to be gaps and require investment. Community engagement mechanisms need to be strengthened and institutionalised to ensure peoples' voices are actually heard and considered in implementation of QOC. To advance the development and implementation of QOC at scale, we encourage other countries to use the LALA framework to build and strengthen health systems that enable QOC, and actively collaborate and contribute to the QOC local learning systems and to the global learning network.

## Data Availability

The data analyzed in this study is subject to the following licenses/restrictions: WHO and Ministries of Health hold this data which can be made available on request. Requests to access these datasets should be directed to muzigabam@who.int.
